# Special Issue: Single Molecule Techniques

**DOI:** 10.3390/molecules20057772

**Published:** 2015-04-28

**Authors:** Hans H. Gorris

**Affiliations:** Institute of Analytical Chemistry, Chemo- and Biosensors, University of Regensburg, 93040 Regensburg, Germany; E-Mail: hans-heiner.gorris@ur.de; Tel.: +49-941-943-4015; Fax: +49-941-943-4064

**Keywords:** atomic force microscopy (AFM), conformational dynamics, dynamic/static heterogeneity, Förster resonance energy transfer (FRET), fluorescence spectroscopy, single molecules, super-resolution microscopy

## Abstract

Technological advances in the detection and manipulation of single molecules have enabled new insights into the function, structure and interactions of biomolecules. This Special Issue was launched to account for the rapid progress in the field of “Single Molecule Techniques”. Four original research articles and seven review articles provide an introduction, as well as an in-depth discussion, of technical developments that are indispensable for the characterization of individual biomolecules. Fluorescence microscopy takes center stage in this Special Issue because it is one of the most sensitive and flexible techniques, which has been adapted in many variations to the specific demands of single molecule analysis. Two additional articles are dedicated to single molecule detection based on atomic force microscopy.

The high sensitivity as well as the high spatial and temporal resolution makes fluorescence microscopy the first choice for many single molecule applications in the life sciences. Since the inception of fluorescence-based single molecule detection at low temperatures [1,2], progress has heavily relied on efficient background suppression strategies and data analysis tools to extract a fluorescent signal generated by a single molecule from the background noise of the surrounding medium. In particular, Förster resonance energy transfer (FRET) has been used as a nanoscale ruler for measuring dynamic changes in biomolecules without ensemble averaging. The review by the group of Grohmann and Tinnefeld [3] provides a comprehensive guideline for beginners in the field who wish to take their experiments to the single molecule level. The critical steps for a successful single molecule experiment are discussed with a focus on FRET. The reader is also guided through the data analysis and interpretation of various single molecule experiments.

DNA is an important class of biomolecules, which has been investigated by single molecule FRET experiments. As FRET-based single molecule experiments require labeling with a donor and an acceptor dye, it is important to minimize interference of the labels with the biological system to be investigated. The Schlierf group [4] has addressed this important design consideration by investigating the influence of dye labeling on the formation of a DNA hairpin. The Fitter group [5] optimized FRET measurements by analyzing the motions of the dyes attached to double stranded DNA via a linker and developed a new data analysis algorithm to distinguish dye motions from intramolecular changes in DNA.

Proteins are the second class of biomolecules that are covered by this Special Issue. The review by the Hatzakis group [6] introduces the most important single molecule techniques that have been used to get new insights into the structure and function of proteins. Again FRET is an indispensable tool for observing conformational changes in single protein molecules. The group of Canters [7] describes the design of single molecule FRET experiments for investigating energy transfer processes in oxidoreductases. Single enzyme molecules can also be analyzed with respect to their individual substrate turnover rates if suitable fluorogenic substrates are available. Our review [8] presents different approaches for enclosing many single enzyme molecules in very small reaction chambers to observe their individual catalytic activity in parallel.

*In vivo* studies of single fluorescently labeled biomolecules in the complex environment of a living cell pose further experimental challenges. The review of the Biteen group [9] aims to guide the reader into single molecule super-resolution imaging and tracking in bacterial cells whereas the Taniguchi group [10] focuses on the analysis of single cell protein expression. Calderon [11] presents an advanced data analysis technique to detect dynamic state changes of single molecules in the intrinsically noisy environment of a cell.

This special issue is completed by two contributions on atomic force spectroscopy (AFM). The review of the Keller group [12] describes the use of DNA origami as a scaffold for positioning biomolecules with high precision that can then be probed by AFM. The work of the Ebner group [13] highlights AFM as a technique that—unlike fluorescence microscopy—can both visualize and manipulate single molecules by exerting minute mechanical forces.

Finally, I wish to thank all authors for their important contributions to this Special Issue. I also thank the staff members of the MDPI editorial office, in particular Ms. Jessica Bai, for their support.
